# A multicenter study on the effect of continuous hemodiafiltration intensity on antibiotic pharmacokinetics

**DOI:** 10.1186/s13054-015-0818-8

**Published:** 2015-03-13

**Authors:** Darren M Roberts, Xin Liu, Jason A Roberts, Priya Nair, Louise Cole, Michael S Roberts, Jeffrey Lipman, Rinaldo Bellomo, On behalf of the RENAL Replacement Therapy Study Investigators

**Affiliations:** Burns Trauma and Critical Care Research Centre, The University of Queensland, Level 3 Ned Hanlon Building, Royal Brisbane and Women’s Hospital, Butterfield Street, Brisbane, Queensland 4029 Australia; Therapeutics Research Centre, School of Medicine, University of Queensland, Princess Alexandra Hospital, Ipswich Road, Brisbane, Queensland 4102 Australia; University of South Australia, City East Campus, GPO Box 2471, Adelaide, South Australia 5000 Australia; The Queen Elizabeth Hospital, 28 Woodville Road, Woodville South, Adelaide, South Australia, 5011 Australia; Department of Intensive Care Medicine, Level 3 Ned Hanlon Building, Royal Brisbane and Women’s Hospital, Butterfield Street, Brisbane, Queensland 4029 Australia; Intensive Care Unit, St Vincent’s Hospital, Victoria Street, Darlinghurst, NSW 2010 Australia; Intensive Care Unit, Nepean Hospital, Derby Street, Kingswood, NSW 2747 Australia; Department of Intensive Care, Austin Health, 145 Studley Road, Heidelberg, Victoria 3084 Australia

## Abstract

**Introduction:**

Continuous renal replacement therapy (CRRT) may alter antibiotic pharmacokinetics and increase the risk of incorrect dosing. In a nested cohort within a large randomized controlled trial, we assessed the effect of higher (40 mL/kg per hour) and lower (25 mL/kg per hour) intensity CRRT on antibiotic pharmacokinetics.

**Methods:**

We collected serial blood samples to measure ciprofloxacin, meropenem, piperacillin-tazobactam, and vancomycin levels. We calculated extracorporeal clearance (CL), systemic CL, and volume of distribution (Vd) by non-linear mixed-effects modelling. We assessed the influence of CRRT intensity and other patient factors on antibiotic pharmacokinetics.

**Results:**

We studied 24 patients who provided 179 pairs of samples. Extracorporeal CL increased with higher-intensity CRRT but the increase was significant for vancomycin only (mean 28 versus 22 mL/minute; *P* = 0.0003). At any given prescribed CRRT effluent rate, extracorporeal CL of individual antibiotics varied widely, and the effluent-to-plasma concentration ratio decreased with increasing effluent flow. Overall, systemic CL varied to a greater extent than Vd, particularly for meropenem, piperacillin, and tazobactam, and large intra-individual differences were also observed. CRRT dose did not influence overall (systemic) CL, Vd, or half-life. The proportion of systemic CL due to CRRT varied widely and was high in some cases.

**Conclusions:**

In patients receiving CRRT, there is great variability in antibiotic pharmacokinetics, which complicates an empiric approach to dosing and suggests the need for therapeutic drug monitoring. More research is required to investigate the apparent relative decrease in clearance at higher CRRT effluent rates.

**Trial registration:**

ClinicalTrials.gov NCT00221013. Registered 14 September 2005.

**Electronic supplementary material:**

The online version of this article (doi:10.1186/s13054-015-0818-8) contains supplementary material, which is available to authorized users.

## Introduction

Bacterial sepsis is common and increases mortality in critically ill patients with acute kidney injury (AKI) [1]. The administration of antibiotics is a key component of therapy in these patients [2]. The influence of antibiotic concentration on bacterial kill has been determined *in vitro* and is the basis of current approaches to antibiotic dosing [3]. Logically, incorrect dosing secondary to a poor understanding of pharmacokinetics may contribute to adverse outcomes, including treatment failure [4] or the escalation of antibiotic resistance [5].

Key aspects of pharmacokinetics like antibiotic clearance (CL) and volume of distribution (Vd) are altered in critically ill patients with AKI because of loss or renal clearance, volume expansion, and interventions such as vasopressors and continuous renal replacement therapy (CRRT) [6,7]. Moreover, extracorporeal CL during CRRT is influenced by the physicochemical and pharmacokinetic properties of the antibiotic, blood flow, dialysate flow, and ultrafiltration rate and by membrane fouling and filter clotting [8]. These variables may lead to sub-therapeutic blood concentrations and contribute to treatment failure [9-11]. However, data on antibiotic pharmacokinetics during CRRT are limited in scope and detail [12], making correct prescription problematic. Accordingly, in patients treated with CRRT of different intensities, we aimed to evaluate variability in CL and Vd and to assess the effect of the CRRT prescription on extracorporeal and systemic antibiotic CL and Vd.

## Methods

### Clinical

This was a nested cohort prospective multicenter observational pharmacokinetic study within a large randomized controlled trial of CRRT intensity (trial registration: ClinicalTrials.gov NCT00221013, registered 14 September 2005) [13]. Inclusion criteria for this study, known as the Randomized Evaluation of Normal vs. Augmented Level of CRRT (RENAL) study, have been previously published and are outlined in the online supplement (Additional file 1).

In brief, patients were randomly assigned to receive post-dilutional hemodiafiltration as either a higher (40 mL/kg body weight/hour effluent flow rate) or lower (25 mL/kg body weight/hour effluent flow rate) intensity rate using equal size filters with polyacrylonitrile membranes. By protocol, the target prescribed effluent flow was achieved through an equal contribution of dialysate flow and ultrafiltration. Blood flow was 200 mL/minute for all study patients except two (150 or 180 mL/minute).

Four hospitals in three separate geographical regions participated in this study. Institutional review boards approved the study, and informed consent and demographic, clinical, and laboratory data were obtained [13]; see the online supplement (Additional file 1) for more details.

Pharmacokinetic sampling occurred at three time points each day: (1) immediately before antibiotic dosing, (2) after completion of their intravenous infusion, and (3) at 4 hours after completion of infusion. A patient could participate on more than one occasion but on different days. The time of sampling was recorded exactly, and at each time point, a pre-filter blood sample and CRRT effluent sample were obtained simultaneously. Plasma was separated by centrifugation, immediately frozen, and stored at −70°C until analysis.

### Laboratory techniques

The plasma and effluent concentrations of ciprofloxacin (molecular weight (MW) 331.3), meropenem (MW 383.5), piperacillin (MW 517.6), and tazobactam (MW 300.3) were determined by using a validated liquid chromatography with tandem mass spectrometry method that has been described previously [9]; see online supplement (Additional file 1) for more details. The plasma concentrations of vancomycin (MW 1449.3), urea (MW 60.1), and creatinine (MW 113.1) were determined by using commercial assays by the Chemical Pathology Laboratory, Princess Alexandra Hospital, Queensland Health Scientific and Forensic Services, Brisbane, Australia.

### Pharmacokinetic calculations

The antibiotic CL from hemodiafiltration (CL_HDF_) was calculated at each time point on the basis of drug recovery in the CRRT effluent by using a standard formula [8,14]:

$$ {\mathrm{C}\mathrm{L}}_{\mathrm{HDF}} = \kern0.37em \left({\mathrm{C}}_{\mathrm{E}}/\;{\mathrm{C}}_{\mathrm{P}}\right)\times {\mathrm{Q}}_{\mathrm{E}}, $$

where C_E_ is the solute concentration in CRRT effluent, Q_E_ the prescribed CRRT effluent flow, and C_P_ the solute concentration in plasma. The C_E_/C_P_ ratio is referred to as effluent or dialysate saturation [14] or the saturation coefficient (Sd) [8]. The Sd is the diafiltration equivalent of sieving coefficient, which is calculated in the same way during hemofiltration. The Sd was also calculated for urea and creatinine as a measure of filter membrane function.

Using pre-filter plasma concentrations at the three time points, the systemic CL (CL_s_) and Vd were calculated. These time points were chosen so that the first and second points related to Vd and the second and third related to CL_s_. The antibiotic plasma concentration-time data were fitted to one-, two-, or three-compartment models by non-linear mixed-effects population modelling (NONMEM software version 6.1; GloboMax LLC, Hanover, MD, USA) [15]. A Digital Fortran compiler was used, and runs were executed by using Wings for NONMEM (WFN) version 616.

Data were analyzed by using the first-order conditional estimation method with interaction. Between-subject variability was calculated by using an exponential variability model. Residual unexplained variability was tested by using exponential or additive random error or both. Visual inspection of diagnostic scatter plots and the NONMEM objective function value (OFV) were used to evaluate goodness of fit. Statistical comparison of nested models was undertaken in the NONMEM program on the basis of a chi-square test of the difference in OFV. A decrease in the OFV of 3.84 units (*P* <0.05) was considered statistically significant.

The influence of the properties of a given antibiotic was assessed by using meropenem and vancomycin because they were the most commonly used antibiotics in this series and differed in terms of size (MW 438 versus 1,486 Daltons, respectively) and protein binding (less than 10% versus 30%, respectively [6]).

The systemic elimination half-life (T_1/2_) was calculated as follows:

$$ {\mathrm{T}}_{1/2} = \kern0.37em \left(0.693\times \mathrm{V}\mathrm{d}\right)/{\mathrm{CL}}_{\mathrm{s}} $$

The proportion of CL_s_ attributed to hemodiafiltration was calculated by:

$$ \%\mathrm{C}\mathrm{L}\kern0.37em =\kern0.37em \left({\mathrm{CL}}_{\mathrm{HDF}}/{\mathrm{CL}}_{\mathrm{s}}\right)\times 100. $$

In the case of CL_HDF_ being determined on multiple occasions in the same patient during the time period when CL_s_ was calculated, %CL was determined by using the mean CL_HDF_.

### Statistical calculations

Statistical analyses were conducted according to the normality of data which was determined by using the D’Agostino and Pearson omnibus normality test. Non-parametric data were subject to the Mann-Whitney and Spearman r correlation tests, whereas parametric data were compared by using the Student *t* test (with Welch’s correction when variances were not equal) and Pearson r correlation test. The goodness of fit (r^2^) of statistically significant correlations was determined by using linear regression. All regressions and statistics were conducted by using GraphPad Prism version 5.03 for Windows (GraphPad Software, San Diego, CA, USA), and a *P* value of less than 0.05 was considered statistically significant.

## Results

Sampling occurred at 179 time points in 24 patients: ciprofloxacin (19), meropenem (65), piperacillin-tazobactam (29), and vancomycin (66). The demographics, baseline clinical characteristics, and other treatments for the 24 study patients have been presented previously [9]. Briefly, mean age ± standard deviation was 62 ± 17 years, most were men (16:8), and mean weight was 82 ± 17 kg. Thirteen patients were diagnosed with sepsis, and edema was present in 12 patients. Admission APACHE (Acute Physiology and Chronic Health Evaluation) III and SOFA (Sequential Organ Failure Assessment) scores were 103 ± 17 and 11.0 ± 3.0, respectively. Ventilatory support was required in 75% of patients, and 90-day mortality was 42%. The serum creatinine concentration at randomization was 273 ± 123 μmol/L, and oligo-anuria was present in eight patients. The prescribed effluent flow rates in the higher- and lower-intensity groups were a median (interquartile range, IQR) of 2,800 (2,600 to 3,100) mL/hour and 2,130 (2,000 to 2,375) mL/hour, respectively (*P* <0.0001).

### Extracorporeal antibiotic removal

The antibiotic pre-filter plasma concentration correlated with the CRRT effluent concentration for each of the antibiotics (online supplement Figure S1; see Additional file 1), and Sd was less than 1.0 in 94% of samples. The influence of CRRT intensity on antibiotic CL_HDF_ is shown in Table 1. In general, CL_HDF_ increased with higher-intensity CRRT, but this effect was significant for vancomycin only (mean 28 versus 22 mL/minute; *P* = 0.0003, Student *t* test). Wide standard deviations or IQRs were noted in each case (Table 1). A correlation was noted between the prescribed effluent flow rate and CL_HDF_ for vancomycin but not for other antibiotics (online supplement Figure S2; see Additional file 1).

**Table 1:** **The effect of continuous renal replacement therapy intensity on extracorporeal antibiotic clearance**

**Antibiotic**		**Antibiotic clearance, higher intensity**	**Antibiotic clearance, lower intensity**	***P*** **value**
Ciprofloxacin	Median (IQR)	19 (13-24); n = 12	17 (16-20); n = 7	0.5139
	Mean (SD)	19 ± 8	17 ± 3	
Meropenem	Median (IQR)	23 (16-29)	21 (15-28)	0.4802^a^
	Mean (SD)	23 ± 13; n = 35	21 ± 9; n = 28	
Piperacillin	Median (IQR)	22 (21-31)	24 (17-31)	0.9091^a^
	Mean (SD)	25 ± 10; n = 11	26 ± 12; n = 17	
Tazobactam	Median (IQR)	37 (34-49)	56 (41-66)	0.0642^a,b^
	Mean (SD)	38 ± 13; n = 11	53 ± 24; n = 17	
Vancomycin	Median (IQR)	28 (24-33)	21 (19-25)	<0.0001^a,b^
	Mean (SD)	28 ± 7; n = 35	22 ± 5; n = 31	

^a^Statistical significance was determined by using the *t* test because the data were considered normally distributed; the Mann-Whitney test was used for the remaining analyses. ^b^ *P* <0.05. IQR, interquartile range; SD, standard deviation.

The mean CL_HDF_ of meropenem and vancomycin did not differ significantly: higher intensity 23 ± 13 versus 28 ± 7 mL/minute (*P* = 0.0610) and lower intensity 22 ± 5 versus 21 ± 9 mL/minute (*P* = 0.6899), respectively. The CL_HDF_ of piperacillin was less than that of tazobactam, such that the median CL_HDF_ ratio of piperacillin to tazobactam was 0.56 (IQR 0.39 to 0.72). The median ratio of the plasma concentrations of piperacillin to tazobactam was 14.9 (IQR 10.7 to 23.2, range 7.5 to 41.7). The Sd of vancomycin and meropenem was not consistently influenced by the albumin concentration or hematocrit (online supplement Figure S3; see Additional file 1).

### Extracorporeal uremic solute removal

**Figure 1:**
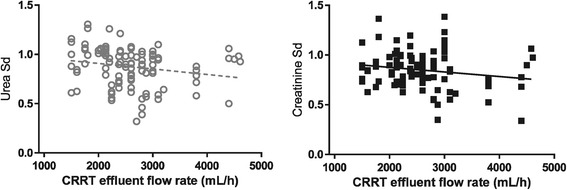
**Marked variability but negative association between the saturation coefficients (Sd) of urea (significant) and creatinine (non-significant) and CRRT effluent flow rate.** Urea: Pearson r = −0.2016, *P* = 0.0383, r^2^ = 0.04063, n = 106 pairs; creatinine: Pearson r = −0.1790, *P* = 0.0704, n = 103 pairs. CRRT, continuous renal replacement therapy.

**Figure 2:**
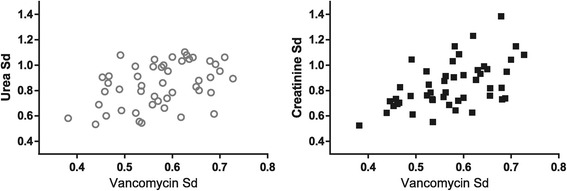
**Positive correlation between the saturation coefficient (Sd) of vancomycin with both urea and creatinine.** Urea: Spearman r = 0.3817, *P* = 0.0074, n = 48 pairs; creatinine: Spearman r = 0.5297, *P* = 0.0001, n = 48 pairs.

Urea CL_HDF_ was significantly higher in the higher-intensity group (mean 41 ± 14 versus 33 ± 7 mL/minute, *P* = 0.0002); the same was observed for creatinine CL_HDF_ (mean 40 ± 13 versus 31 ± 7 mL/minute, *P* = 0.0003). A correlation was noted between the prescribed effluent flow rate and CL_HDF_ for urea and creatinine (online supplement Figure S4; see Additional file 1). However, despite the lack of correlation between effluent flow rate and creatinine Sd, a negative correlation was noted with urea Sd and effluent flow rate (Figure 1). Moreover, the urea and creatinine Sd varied markedly within comparable effluent flow rates. A significant association between the Sd of urea, creatinine, meropenem, or vancomycin with the age of CRRT filter was not observed (online supplement Figure S5; see Additional file 1). A positive and significant correlation was noted with the Sd of both urea and creatinine for vancomycin (Figure 2) but not meropenem (online supplement Figure S6; see Additional file 1).

### Systemic clearance and volume of distribution

The results of the population pharmacokinetic model development and evaluation are outlined in the online supplement (Additional file 1). The estimates of CL_s_ and Vd show that inter-individual differences were significant for CL_s_—coefficient of variation (CV%) 10.0 for ciprofloxacin and 52.3 for tazobactam—and Vd (CV% 24.9 to 37.8) (Table 2).

**Table 2:** **Calculated systemic pharmacokinetic parameters for antibiotics and tazobactam assessed in this study compared with values reported for critically ill patients in the literature**

	**Population estimate in this study (and range in individuals)**		**Random effects – BSV (CV%)**		**Random error**		**Values reported in the literature**	
	**CL, mL/minute**	**Vd**	**BSV**_**CL**_	**BSV**_**Vd**_	**RUV (CV%)**	**SD, mg/L**	**CL, mL/minute** ^**a**^	**Vd, L** ^**a**^
Ciprofloxacin	58 (53-63)	37.7 L	10.0	34.6	-	0.99	34-617 [16-18]	28-224 [16-18]
		(0.32-0.88 L/kg)						
Meropenem	38 (23-95)	17.5 L	34.5	37.8	16.8	2.07	23-236 [10,19-27]	12-212 [10,19-27]
		(0.14-0.61 L/kg)						
Piperacillin	59 (37-115)	18.7 L	40.9	27.2	44	-	24-438 [10,21,28-34]	10-120 [10,21,28-34]
		(0.14-0.29 L/kg)						
Tazobactam	113 (45-248)	49.3 L	52.3	34.5	41	-	22-180 [28-30,32,34]	8-60 [28-30,32,34]
		(0.54-0.55 L/kg)						
Vancomycin	25 (16-33)	39.7 L	27.4	24.9	16	0.032	23-73 [35-37]	20-137 [35-37]
		(0.32-0.74 L/kg)						

^a^Calculated on the basis of the mean body weight reported in the study or using 70 kg, if required. BSV, between-subject variability; CL, clearance; CV, coefficient of variation; RUV, residual unexplained variability; SD, standard deviation; Vd, volume of distribution.

**Figure 3:**
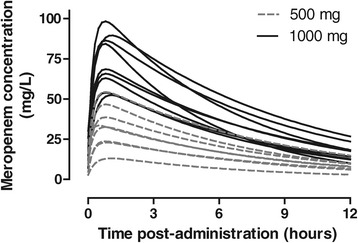
**Marked inter-individual and inter-occasion variability in the plasma concentration-time profile of meropenem over 12 hours following 17 doses of either 500 or 1,000 mg to 12 patients.** Simulated concentration-time profiles using a single compartment equation are based on the trough concentration, time of infusion, and calculated systemic clearance and volume of distribution for each occasion (performed by using GraphPad Prism version 4.03 for Windows; GraphPad Software, San Diego, CA, USA).

The net effect of this variability is shown by using simulation in the case of meropenem, in which an approximate dose-response relationship is observed, but despite a twofold difference in dose, the concentration-time curves are not clearly separated (Figure 3).

In the case of vancomycin and meropenem, the prescribed CRRT effluent rate did not significantly influence CL_s_ (online supplement Figure S7; Additional file 1), Vd, or elimination half-life (data not shown).

### Relationship between systemic and extracorporeal clearance

The contribution of CL_HDF_ to the calculated CL_s_ also varied widely. The medians and IQRs were ciprofloxacin 32% (29% to 34%), meropenem 66% (42% to 76%), piperacillin 23% (19% to 27%), tazobactam 49% (39% to 57%), and vancomycin 92% (84% to 109%).

### Inter-occasional pharmacokinetic variability

**Figure 4:**
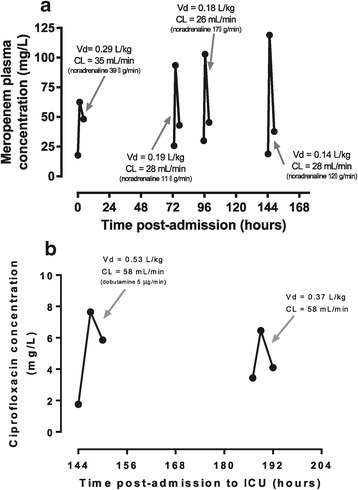
**Examples of inter-occasion variability in antibiotic pharmacokinetics. (a)** Admission to the intensive care unit (ICU) after coronary artery bypass grafts and valve repair, requiring mechanical ventilation and vasopressors for hypotension (not attributed to sepsis). This patient received higher-intensity continuous renal replacement therapy (CRRT) and did not survive to hospital discharge. Meropenem (1 g) was administered on each occasion. **(b)** Admission to the ICU from the emergency department and subsequently diagnosis with *Klebsiella* sepsis associated with a soft tissue infection, complicated by edema and hypoalbuminemia (24 g/L). Treatment included mechanical ventilation, antibiotics, and dopamine infusion (decreasing requirements during admission, not required at the time of second samples). This patient received higher-intensity CRRT and was alive at 90 days post-admission. Ciprofloxacin (200 mg) was administered on both occasions. CL, clearance; Vd, volume of distribution.

The concentration-time profile, clinical information, and pharmacokinetic parameters for two of the patients who provided samples on multiple days are shown in Figure 4. Much day-to-day variability in antibiotic pharmacokinetics was noted, although each patient received a fixed dosage of CRRT.

## Discussion

### Key findings

In this multicenter pharmacokinetic study of four antibiotics and tazobactam, the prescribed intensity of CRRT did not adequately predict CL_HDF_ or Sd, CL_s_, Vd, or half-life. This did not appear to be explained by the age of the filter, hematocrit, or serum albumin concentration. A correlation was noted between antibiotic concentration in the effluent and pre-filter plasma samples, where the effluent concentration was lower than that of plasma. Using urea and creatinine as biomarkers of membrane function, despite a correlation between effluent flow and CL_HDF_, we detected failure of equilibration between dialysate and plasma during higher CRRT intensity. The correlation between the Sd of urea or creatinine with antibiotics was inconsistent, limiting their role as a surrogate measures for clearance.

### Principles of extracorporeal solute removal

The influence of components of the prescription of an extracorporeal treatment was recently reviewed [38]. In the context of CRRT, effluent flow is the rate-limiting step because it is slower than blood flow, so clearance is anticipated to positively correlate with effluent flow rate. Furthermore, clearance by convection is dose-dependent and this is the most efficient method of removing larger molecules. Smaller molecules are also removed efficiently by diffusion; however, the effect of diffusion is non-linear at higher effluent flow rates and this may compromise clearance. Post-filter replacement maximizes removal but this may be at the expense of the life of the filter [39].

Our results suggest that these simple and logical relationships may not be observed in clinical practice. In particular, in our study, the effect of prescribed CRRT effluent flow rate on antibiotic clearance was not consistently dose-dependent. Others have reported similar findings, including a higher Sd for beta-2 microglobulin (MW 11,000) with increasing ultrafiltration rates, and have observed that varying the proportion of dialysate and ultrafiltrate flows in hemodiafiltration influences the Sd of different solutes variably [40].

### Factors that may interfere with extracorporeal solute removal

A correlation was noted between CL_HDF_ and effluent flow for vancomycin, creatinine, and urea but not for other antibiotics. These observations, coupled with the decrease in the Sd of urea with increasing effluent flow, indicate that equilibration across the filter is unpredictable and may decrease at higher effluent flow rates.

This implies a dynamic but unpredictable loss of filter membrane function with use and this loss may be due to partial filter clotting or membrane fouling due to higher transmembrane pressures [41,42]. Smaller molecules may be more affected by this process because their clearance is diffusion-dependent [40]. The age of the filter probably failed to have an influence in our study because of selection bias (filters that are better functioning and less prone to clotting will last longer). Finally, even in laboratory-based testing of dialyzers, there is marked scatter of these data because of any of the above-mentioned factors and changes in transit time due to the effect of internal and back filtration of solutes or water or both [43]. Adsorption of antibiotic to the filter may also contribute to the results observed.

### Influence of continuous renal replacement therapy on clearances

The extracorporeal antibiotic and tazobactam CL_s_ observed are consistent with data in the literature [12]; however, we also noted a wide dispersion of results within a single CRRT treatment arm. This had the effect of reducing the clinical and statistical significance of the relatively small differences in concentration that were observed between groups. The reason for this variability requires further investigation and may include differences in renal (and residual function in patients with AKI) and non-renal clearance mechanisms.

The CRRT regimen used in this study was that of hemodiafiltration. In both types of CRRT, only the free (unbound) antibiotic can be cleared. The antibiotics assessed in this study are minimally protein-bound [44]: ciprofloxacin 30% to 40%, meropenem 8%, piperacillin 20-30%, vancomycin 30% to 40%, and tazobactam 20% to 30% (although these values may change in critical illness [26]); so vancomycin CL is predicted to be less than meropenem. Differences in MW can also influence CL in certain CRRT regimens. For example, the partial diffusive CL used in continuous veno-venous hemodiafiltration will only achieve less vancomycin removal than meropenem; however, we noted inconsistencies in this simple assumption, and possible reasons were discussed above.

### Relationship to previous studies

We observed both inter-individual and inter-occasion (intra-individual) variability in pharmacokinetics. Some variability may depend on the clinical characteristics of patients. For example, in sepsis and critical illness, ciprofloxacin CL and Vd are decreased compared with volunteers matched for creatinine CL and weight [18]. Similarly, the Vd and CL of meropenem are lower in critically ill patients with sepsis compared with those with multitrauma, matched for creatinine CL [22]. Finally, the calculated CL and Vd for piperacillin is lower after multiple doses administered (similar to the context of our data [9]) compared with a single dose [31]. Dynamic changes in residual renal function may specifically contribute to these observations.

### Observations with piperacillin and tazobactam

Piperacillin and tazobactam are co-formulated, and so assessment of their individual pharmacokinetics may inform dosage recommendations. Between-subject variation in pharmacokinetics was observed (Table 2), but with tazobactam there was little variability in the weight-normalized Vd of tazobactam. This suggests that its Vd was influenced primarily by body weight rather than the pathophysiology of critical illness. The CL and Vd of tazobactam were higher than those of piperacillin. Some studies have shown a similar relationship [28,30] but others have not [29]. Some studies have quantified piperacillin pharmacokinetics and extrapolated these directly to those of tazobactam [10,21], but this appears to be reliable. We noted marked variability in the ratio of piperacillin to tazobactam plasma concentrations as reported by others [28].

### Contribution of extracorporeal clearance to the total antibiotic clearance

Systemic CL and elimination half-life did not differ according to CRRT dose, and so the CRRT prescription may not be useful for guiding antibiotic prescribing. This has been observed previously [10,22,26]. There is significant non-renal clearance of vancomycin early in the course of AKI, but this decreases with time [45], which may also contribute to the poor relationship. The contribution of extracorporeal CL to systemic CL was variable. This related to the combined effect of the variability in extracorporeal CL and inter-individual and inter-occasion (despite identical CRRT regimens) variability in systemic pharmacokinetics. In many cases, extracorporeal CL accounted for more than 30% of the observed systemic CL for that antibiotic, which is a suggested threshold for adjustment of the dosing regimen. Such variability in CL limits attempts to develop dosing guidelines.

Of note, CL_HDF_ appeared to exceed CL_s_ in some cases. The reason for this is not apparent from our data but may relate to an error with either of these calculated values. For example, it has been noted that the delivered effluent flow rate is frequently lower than the prescribed flow rate, and so the calculation of clearance based on the prescribed effluent rate may overstate the actual clearance [41].

### Strengths and limitations

We evaluated the pharmacokinetics of multiple clinically relevant antibiotics administered to patients as part of a prospective multicenter observational study within a large randomized controlled trial. Multiple measurements were obtained, and similarities were observed between our data and the literature. Our data, however, greatly expand our understanding of CL, Vd, and pharmacokinetics in the setting of CRRT treatment using different CRRT doses and the advantages of randomization and multiple sampling.

Although median extracorporeal CL values were consistent with the literature, the degree of variability was an unexpected finding. All care was taken with the collection of samples by designated staff, subsequent storage, and then analysis using validated methods. However, we cannot exclude that this reflected unmeasured or unknown clinical or mechanical factors occurring during CRRT (for example, an undetected machine error at the time of sampling or a difference between the prescribed and delivered effluent flow rate); however, this also reflects clinical reality. The impact of these factors may have been reduced if clearance was determined from a prolonged collection (over hours) rather than at a point in time, as used in our study.

Our study might be underpowered to detect a true difference in clearance on the basis of CRRT intensity, relating in part, to the selection of patients. The dose ratio of lower- to higher-intensity CRRT effluent is 0.60 per protocol, but in this study the ratio was 0.76 based on the achieved median effluent flow rates. This may narrow the difference between the two groups, although this effect would be lessened by analysis in terms of the actual effluent production rate as performed here rather than according to milliliters per kilogram.

## Conclusions

We have identified marked variability in systemic CL and Vd of multiple antibiotics in critically ill patients with AKI and also the variable influence of CRRT regimen on extracorporeal CL. Taken together, such variability complicates empiric antibiotic prescribing during CRRT. Ongoing research to further explore what factors contribute to this variability is important. In the meantime, drug monitoring may be the most practical method for ensuring that antibiotic therapeutic targets are achieved in critically ill patients receiving CRRT.

## Key messages

- In this multicenter pharmacokinetic study, the prescribed intensity of continuous renal replacement therapy (CRRT) did not adequately predict solute clearance, volume of distribution, half-life, or the saturation coefficient. This did not appear to be explained by the age of the filter, hematocrit, or serum albumin concentration.
- Using urea and creatinine as biomarkers of membrane function, despite a correlation between effluent flow and clearance from hemodiafiltration (CL_HDF_), we detected failure of equilibration between dialysate and plasma during higher CRRT intensity.
- The correlation between the saturation coefficient of urea or creatinine with antibiotics was inconsistent, limiting their role as a surrogate measures for clearance.
- This variable pharmacokinetics complicates empiric antibiotic prescribing during CRRT, and ongoing research is required to determine factors that contribute to this variability.
- In the meantime, drug monitoring may be the most practical method for ensuring that antibiotic therapeutic targets are achieved in critically ill patients receiving CRRT.

## Additional file

Additional file 1:
**Supplementary data.** Inclusion and diagnostic criteria, quantification of beta-lactam antibiotics (laboratory), population pharmacokinetic model development and evaluation, online supplement figures.

## Abbreviations

AKI: acute kidney injury
C_E_: solute concentration in continuous renal replacement therapy effluent
CL: clearance
CL_HDF_: clearance from hemodiafiltration
CL_s_: systemic clearance
C_P_: solute concentration in plasma
CRRT: continuous renal replacement therapy
CV%: coefficient of variation
IQR: interquartile range
MW: molecular weight
NONMEM: non-linear mixed-effects population modelling software
OFV: (NONMEM) objective function value
Sd: saturation coefficient in the effluent or diafiltrate
T_1/2_: systemic elimination half-life
Vd: volume of distribution
