# Supplementary material for: A multicenter study on the effect of continuous hemodiafiltration intensity on antibiotic pharmacokinetics
Source: Crit Care. 2015 Mar 13;19(1):84. doi: 10.1186/s13054-015-0818-8 (PMC4404619; doi:10.1186/s13054-015-0818-8)
Supplement: Additional file 1: — Supplementary data. Inclusion and diagnostic criteria, quantification of beta-lactam antibiotics (laboratory), population pharmacokinetic model development and evaluation, online supplement figures. [file 13054_2015_818_MOESM1_ESM.pdf]

Online Supplement

Inclusion and diagnostic criteria

Patients were recruited to this study if they fulfilled inclusion criteria for the Randomized Evaluation of Normal vs. Augmented Level of CRRT (RENAL) study (ClinicalTrials.gov number NCT00221013, registered September 14, 2005) [1]. These included acute kidney injury (AKI) and at least one of the following: oliguria, hyperkalemia, severe acidemia (pH <7.2), an elevated plasma urea or creatinine concentration, or clinically significant organ edema.

Severe sepsis was defined as a focus of infection and two of the Systemic Inflammatory Response Syndrome (SIRS) criteria within the 24 hours prior to randomisation.

The study was approved by Human and Research Ethics Committees at each of the participating centres, notably Royal Brisbane and Women's Hospital, Alfred Hospital, St Vincent's Hospital (Sydney) and Nepean Hospital. The study was conducted in accordance with the Declaration of Helsinki.

Written informed consent was obtained from the patient or responsible surrogate by means of either a priori or delayed consent (for further details, see [1]). In the case of the responsible surrogate providing consent, the patient was asked to provide consent at a later point, where possible.

Four hospitals in three separate geographical regions participated in this substudy and the antibiotics studied were ciprofloxacin, meropenem, piperacillin (with tazobactam) and vancomycin. Antibiotic dosing was at the discretion of the treating physician. The protocol for CRRT was hemodiafiltration using a high flux membrane with post-dilutional replacement and the target effluent flow was achieved through an equal contribution of dialysate flow and post-filter fluid replacement.

Samples were obtained prior to administration of a dose of antibiotic, then following completion of the intravenous infusion, at approximately 1 and 4 hours, depending on the antibiotic and clinical factors. At each time point, a blood sample was obtained pre-filter and

post-filter simultaneously with a dialysate sample, the plasma was separated, and samples were immediately frozen and stored at -70 ^{o}C until analysis.

Quantification of beta-lactam antibiotics

The LC/MS/MS assay for antibiotics was validated according to FDA "Guidance for Industry: Bioanalytical Method Validation". The assay was validated both in plasma and in dialysate. When doing the real sample analysis, quality control samples were used to evaluate the assay performance. The assay results were accepted if at least four of every six QC samples are within 15% of their respective nominal concentration.

The concentration ranges of the standard curves were 0.002–1 mg/L for piperacillin, 0.004-2 mg/L for ciprofloxacin, 0.008-4 mg/L for meropenem, and 0.2-40 mg/L for tazobactam.

The concentration range of the standard curve was 5-50 mg/L. The coefficients of variation for interassay and intra-assay precision were <10% for all of the assays.

Free (unbound to plasma protein) concentrations were not measured because previous data suggests that the free concentration is reliably calculated in antibiotics with a relatively low degree of protein binding, as noted with those used here [2].

Population pharmacokinetic model development and evaluation

The time-course of each of the antibiotics in plasma was best described by a one-compartment linear model with zero order input of drug. BSV was described for both CL and Vd. The RUV was described using a combined proportional and additive variability model for meropenem and vancomycin, proportional error model for piperacillin and tazobactam and an additive error model for ciprofloxacin. The goodness of fit plots for each final model were evaluated and showed acceptable results in terms of visual or statistical biases for the prediction.

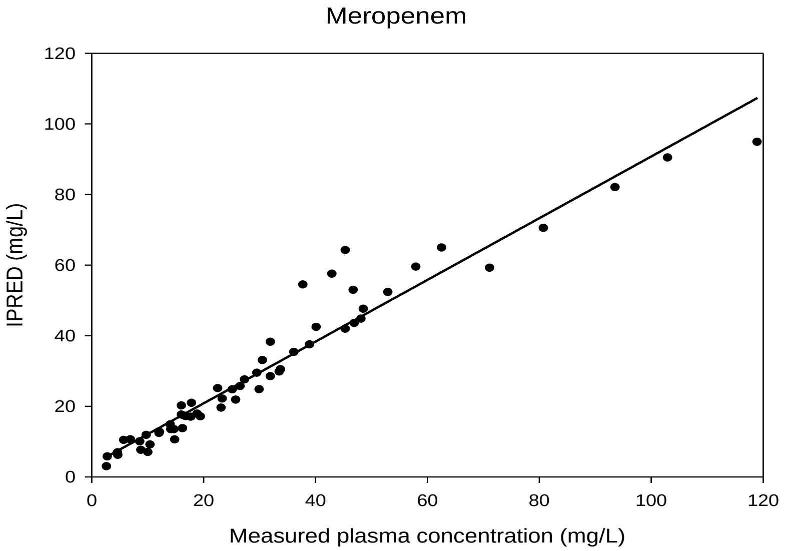

Meropenem
IPRED (mg/L)
Measured plasma concentration (mg/L)
0
20
40
60
80
100
120

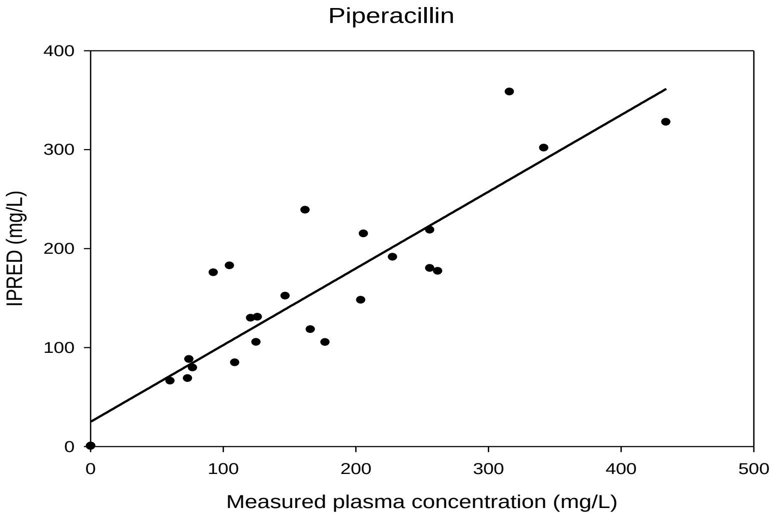

Piperacillin
IPRED (mg/L)
Measured plasma concentration (mg/L)
0
100
200
300
400
500

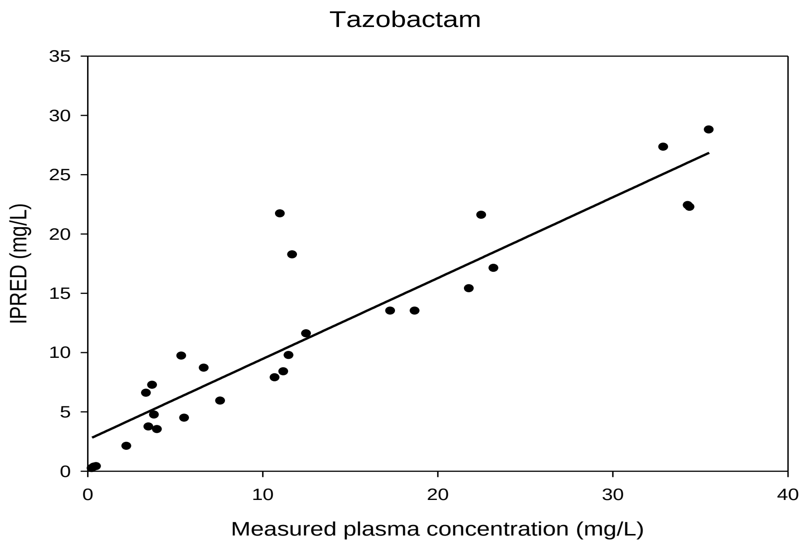
Tazobactam
IPRED (mg/L)
Measured plasma concentration (mg/L)
0
5
10
15
20
25
30
35
0
10
20
30
40

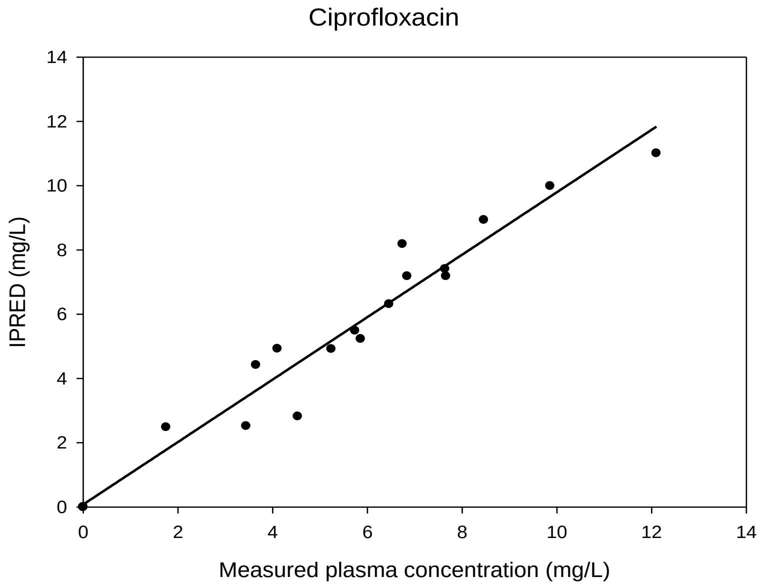
Ciprofloxacin
IPRED (mg/L)
Measured plasma concentration (mg/L)
0
2
4
6
8
10
12
14

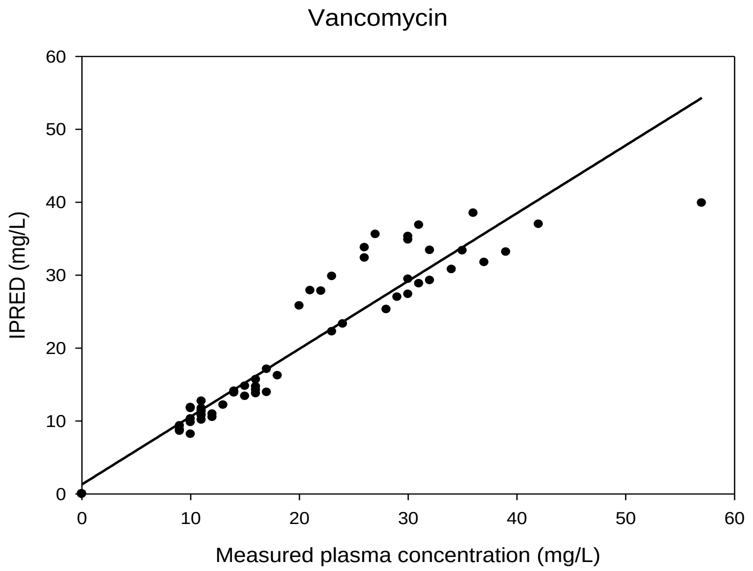
Vancomycin
IPRED (mg/L)
Measured plasma concentration (mg/L)
0
10
20
30
40
50
60

Statistically significant improvements in the base model occurred with inclusion of normalized values of total body weight for meropenem (Vd and CL) and piperacillin (Vd) to calculate the typical value of clearance (TVCL) or typical value of Vd (TVV) based on the weight (WT) of the patient, as follows.

Meropenem: $TVCL = CL*(WT/90.5)^{0.75}$ and $TVV = Vd*(WT/90.5)$

Piperacillin: $TVV = Vd*(WT/77)$

In the final models for ciprofloxacin, tazobactam and vancomycin the TVCL and TVV were the same as CL and Vd for the respective drugs, that is: TVCL = CL and TVV = Vd

Figures

Online supplement Fig 1. Significant correlation between plasma concentration (pre-filter) and dialysate effluent concentration for (a) meropenem, (b) vancomycin, (c) piperacillin, (d) tazobactam, and (e) ciprofloxacin.

(a) Meropenem. Spearman r = 0.8113, P < 0.0001

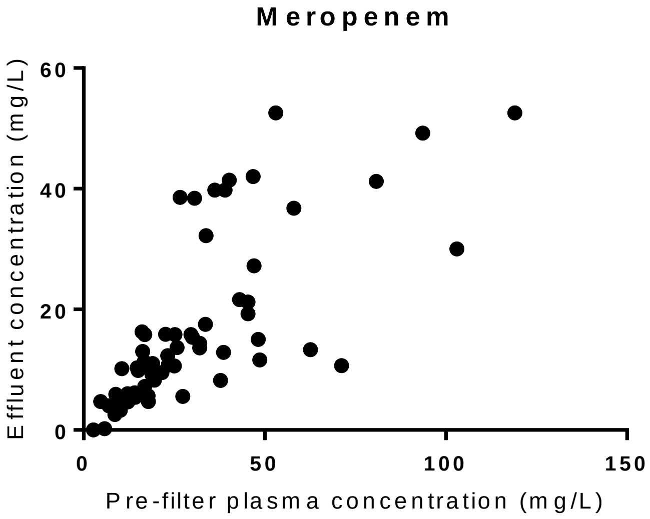

(b) Vancomycin. Spearman r = 0.9269, P < 0.0001

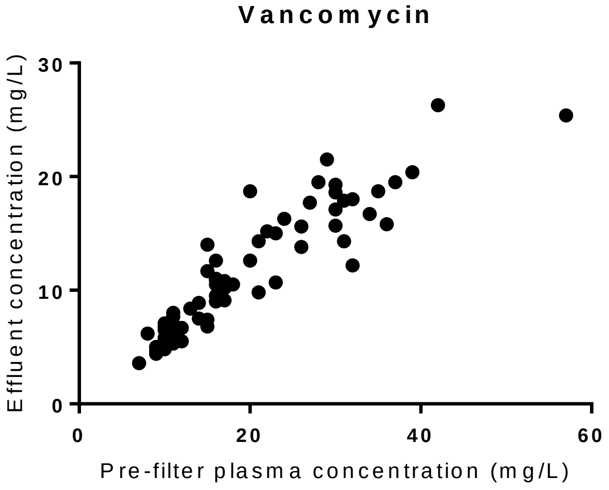

(c) Piperacillin. Spearman r = 0.8853, P < 0.0001

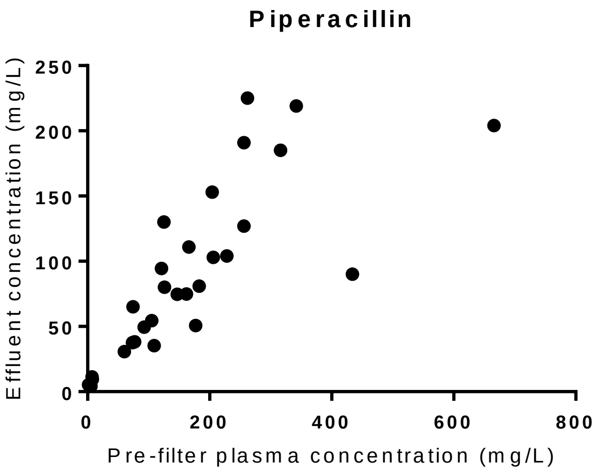

(d) Tazobactam. Pearson r = 0.8123, r^2 = 0.6599, P < 0.0001

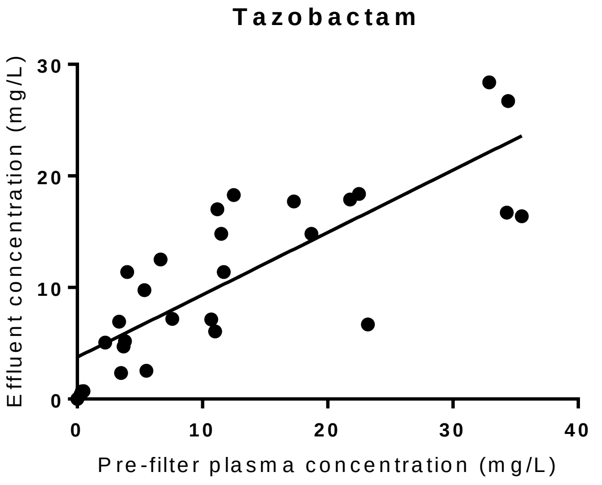

(e) Ciprofloxacin. Spearman r = 0.7411, P = 0.0001

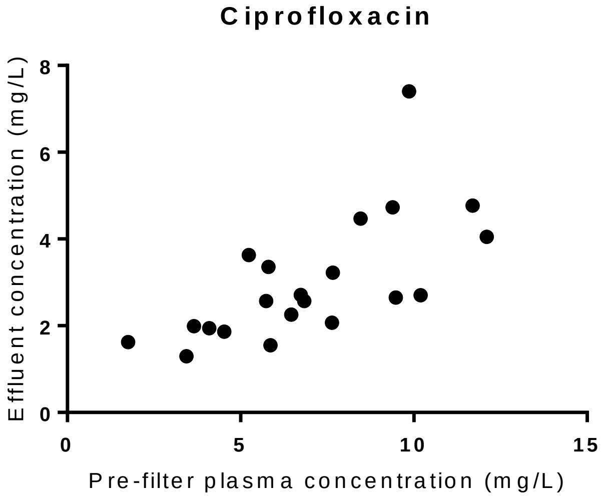

Online supplement Fig 2. Correlation between prescribed dialysate effluent flow rate and extracorporeal clearances of vancomycin, meropenem, urea and creatinine

(a) Vancomycin, positive correlation. Spearman r= 0.6843, P < 0.0001

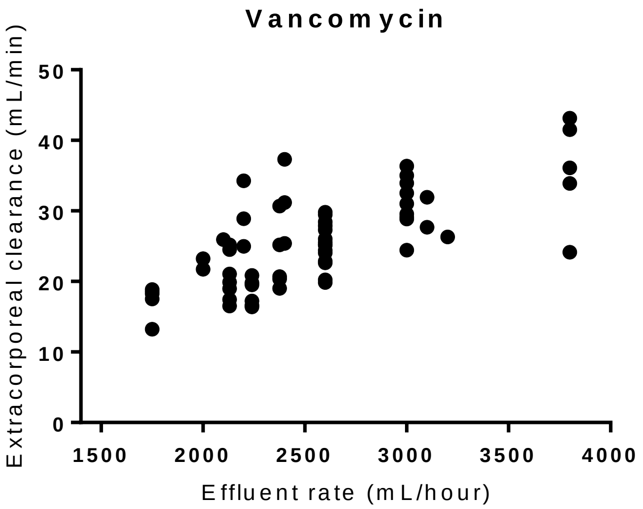

(b) Meropenem, no correlation. Pearson r = 0.1355, r^2 = 0.01836, P = 0.2897

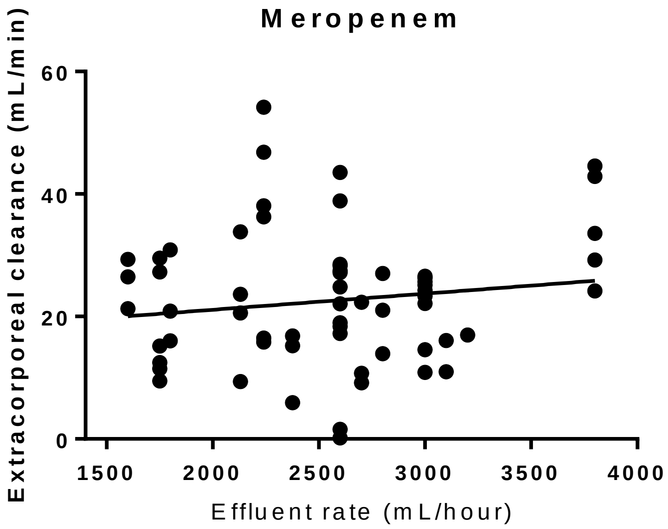

(c) Ciprofloxacin, no correlation. Spearman r= 0.4190, P = 0.0741

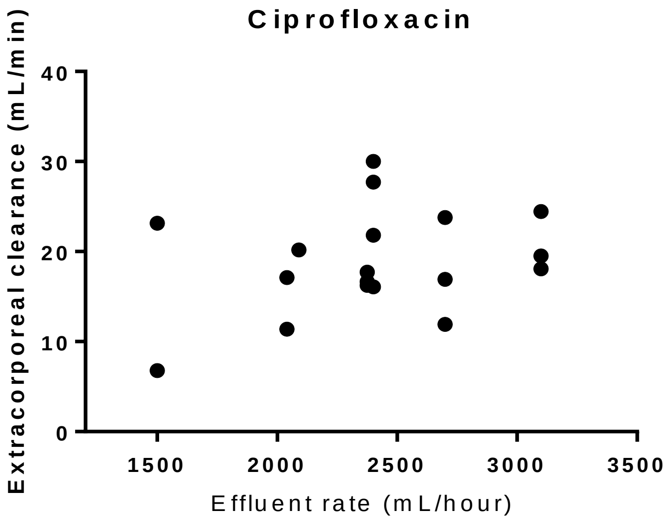

(d) Piperacillin and tazobactam, no correlation. Piperacillin Spearman r = 0.1767, P = 0.3685; Tazobactam Spearman r = -0.03456, P = 0.8614

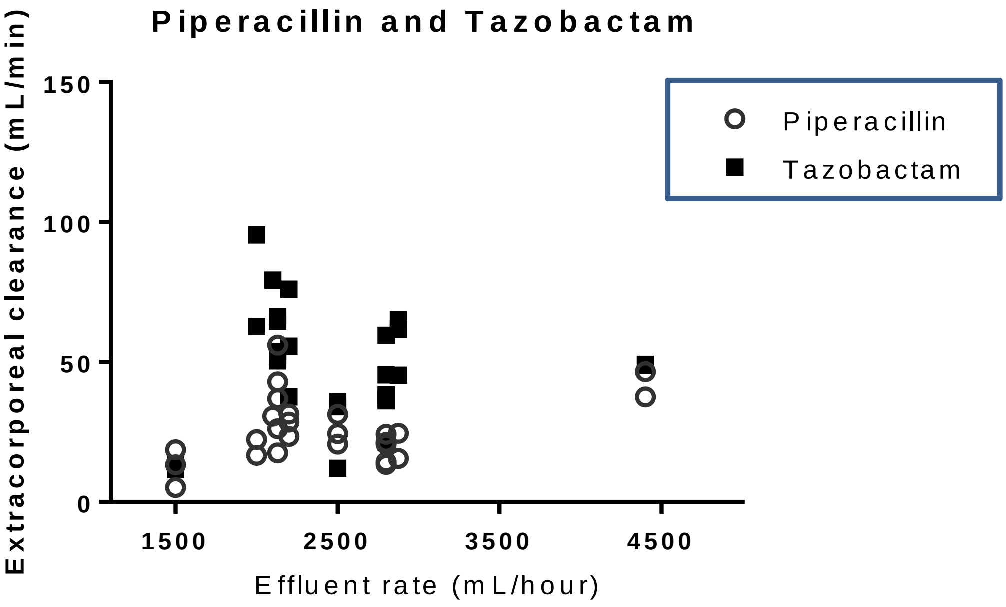

Online supplement Fig 3. Lack of influence of serum albumin concentration or haematocrit on the saturation coefficient of vancomycin* (protein binding 30-60%) and meropenem#.

(a) Serum albumin

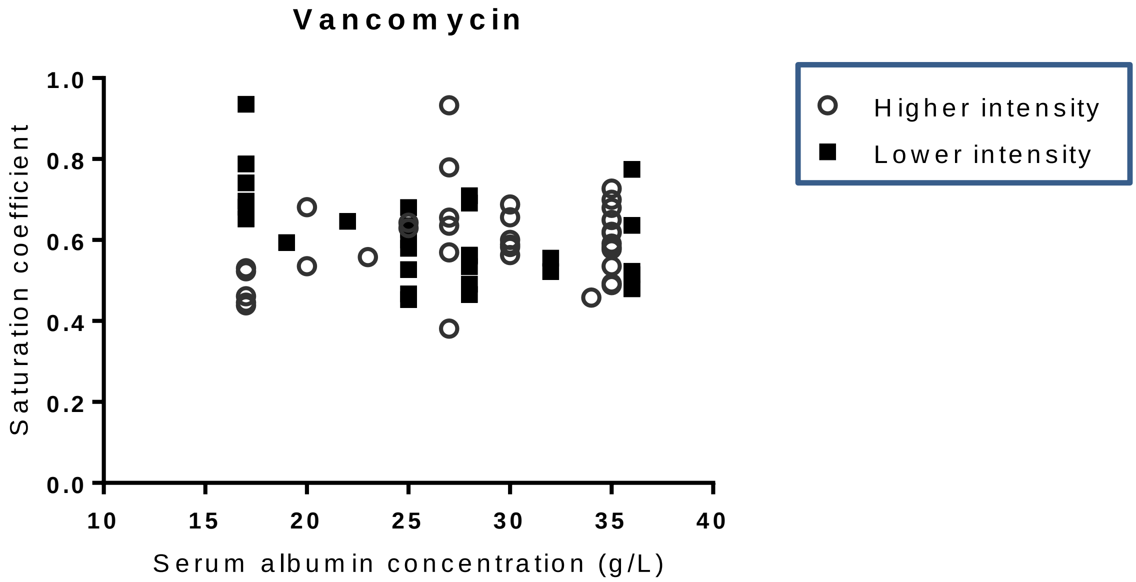

* Higher intensity: Spearman r = 0.2693, P = 0.1178; Lower intensity: Spearman r = -0.4980, P = 0.0044

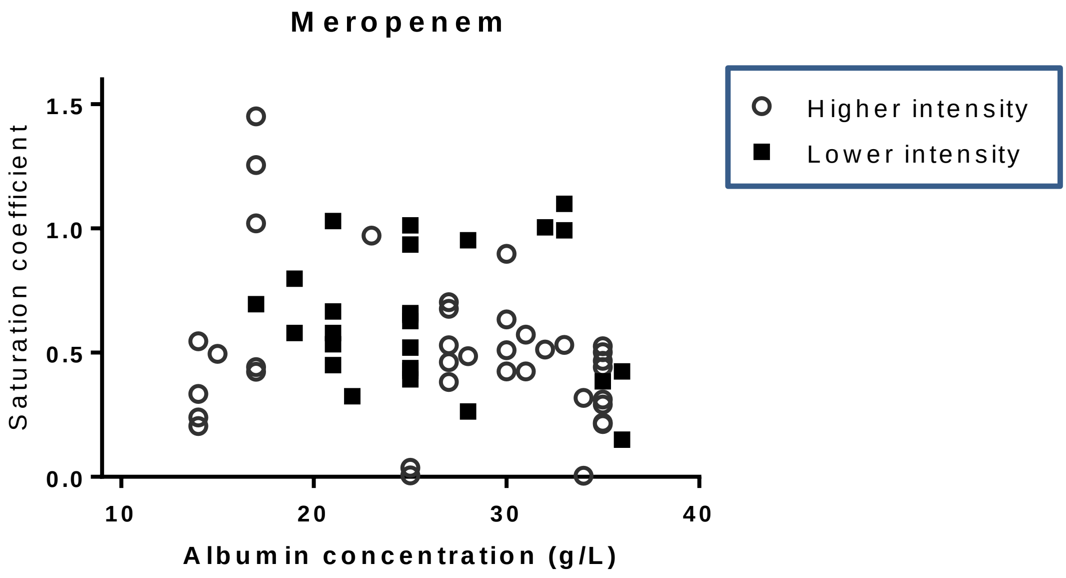

Higher intensity: Spearman r = -0.1675, P= 0.3216; Lower intensity: Spearman r = -0.1582, P = 0.4307

(b) Hematocrit

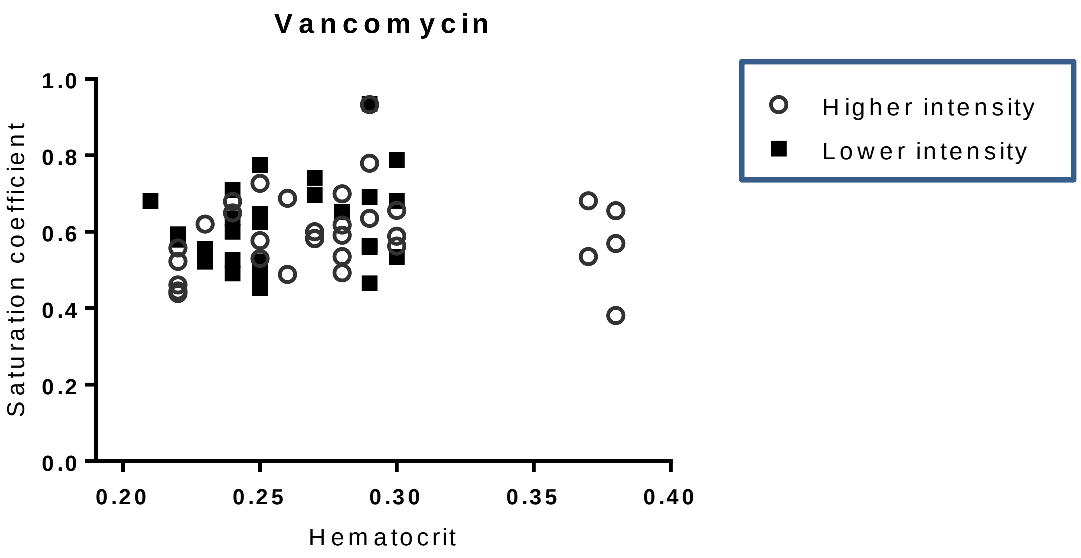

* Higher intensity: Spearman r = 0.2526, P = 0.1631; Lower intensity: Spearman r = 0.2172, P = 0.2405

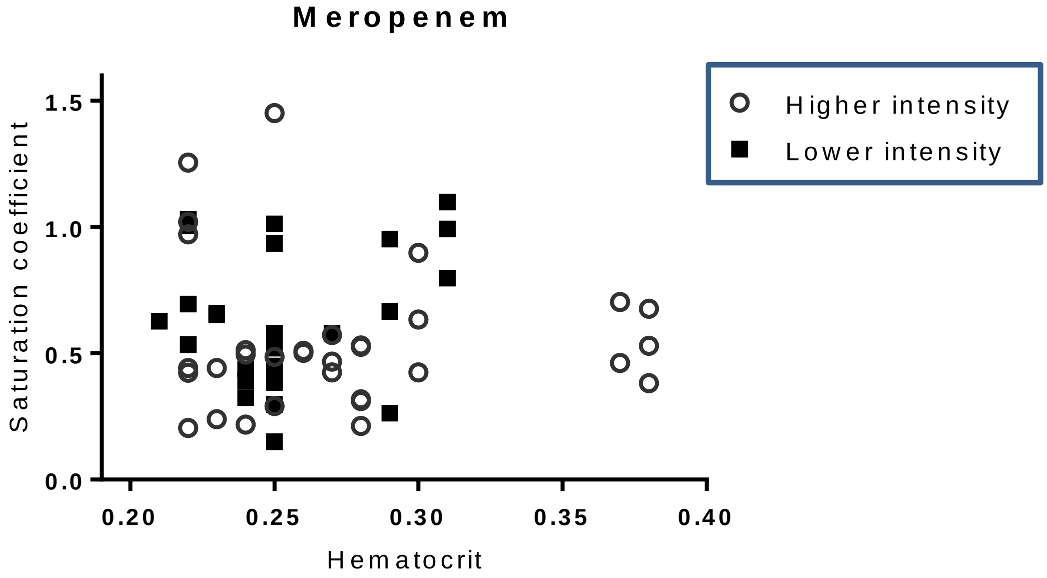

Higher intensity: Spearman r = 0.08067, P = 0.6607; Lower intensity: Spearman r = 0.04475, P = 0.8211

Online supplement fig 4. Positive correlation of urea and creatinine extracorporeal clearance with prescribed effluent flow. Urea: Spearman r = 0.5960, p < 0.0001; and creatinine: r =0.6382, p < 0.0001

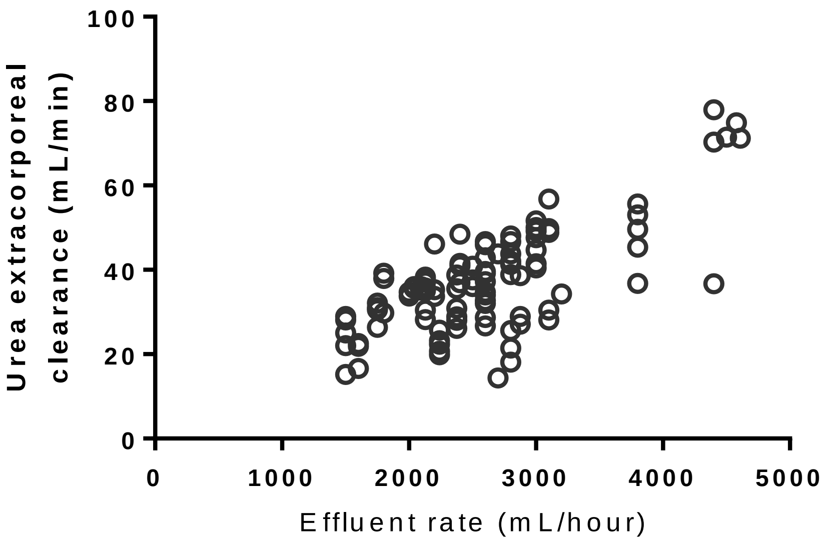

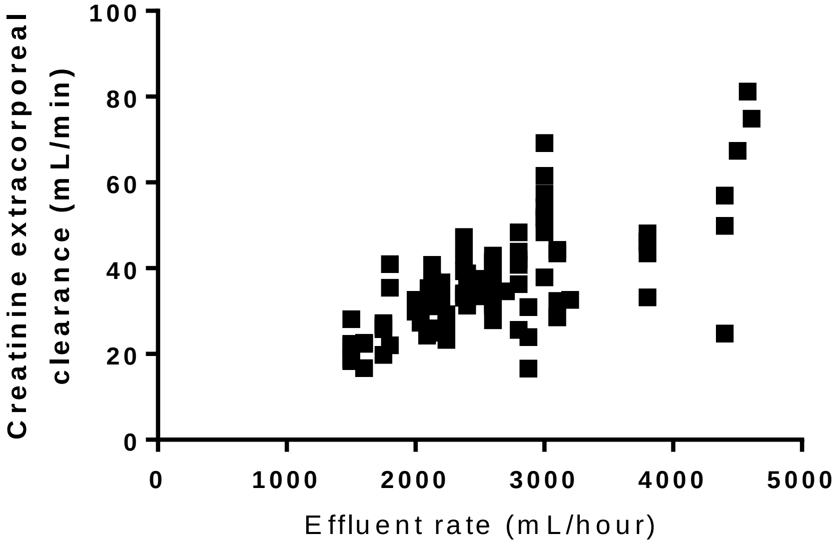

Online supplement fig 5. No correlation between the saturation coefficient (Sd) of urea, creatinine, meropenem or vancomycin with the age of the filter.

Linear graphs

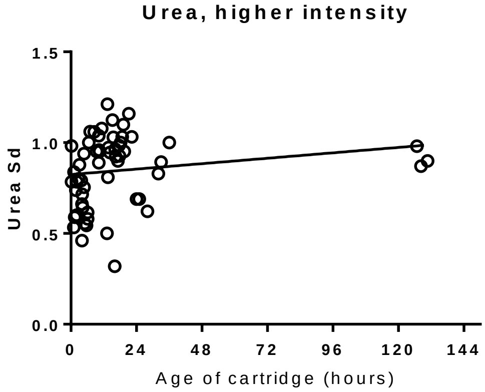

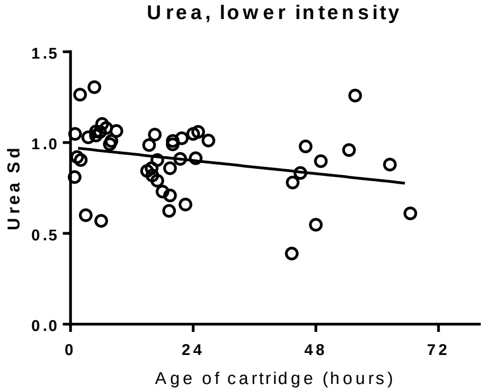

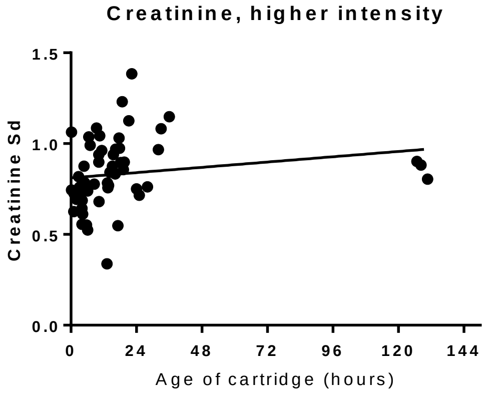

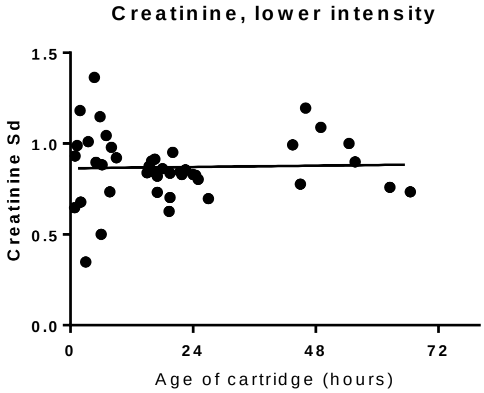

	Age of filter (hours) vs. urea Sd (higher)	Age of filter (hours) vs. creatinine Sd (higher)	Age of filter (hours) vs. urea Sd (lower)	Age of filter (hours) vs. creatinine Sd (lower)
Pearson r	0.1744	0.1775	-0.2794	0.02955
95% confidence interval	-0.08550 to 0.4121	-0.08466 to 0.4167	-0.5245 to 0.008447	-0.2663 to 0.3203
r^2	0.03042	0.03151	0.07806	0.0008733
P value (two-tailed)	0.1865	0.1825	0.0572	0.8472
Number of XY Pairs	59	58	47	45

Semi-log transformed graphs

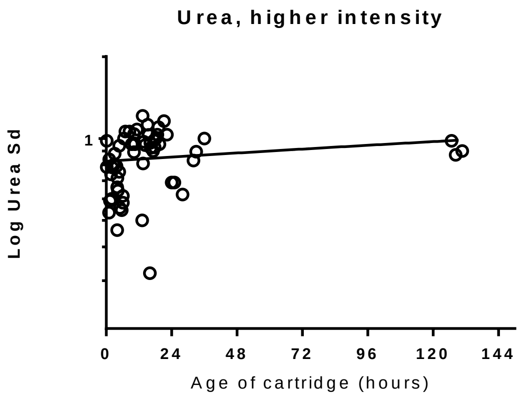

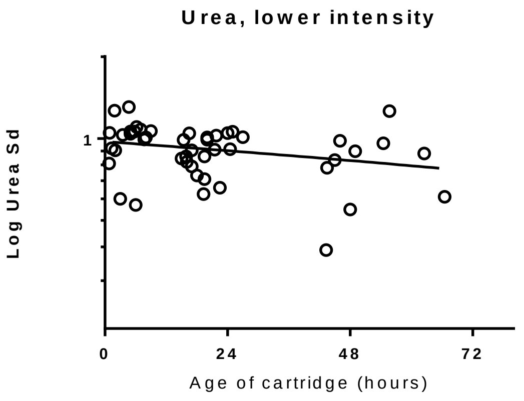

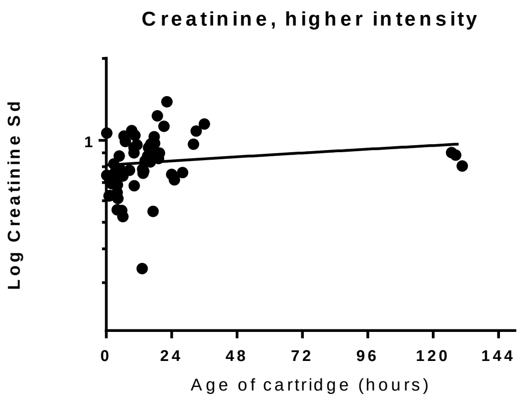

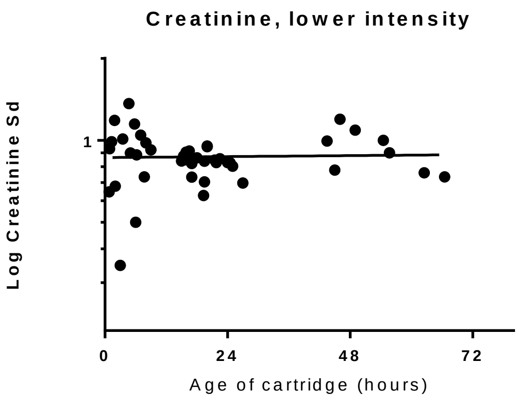

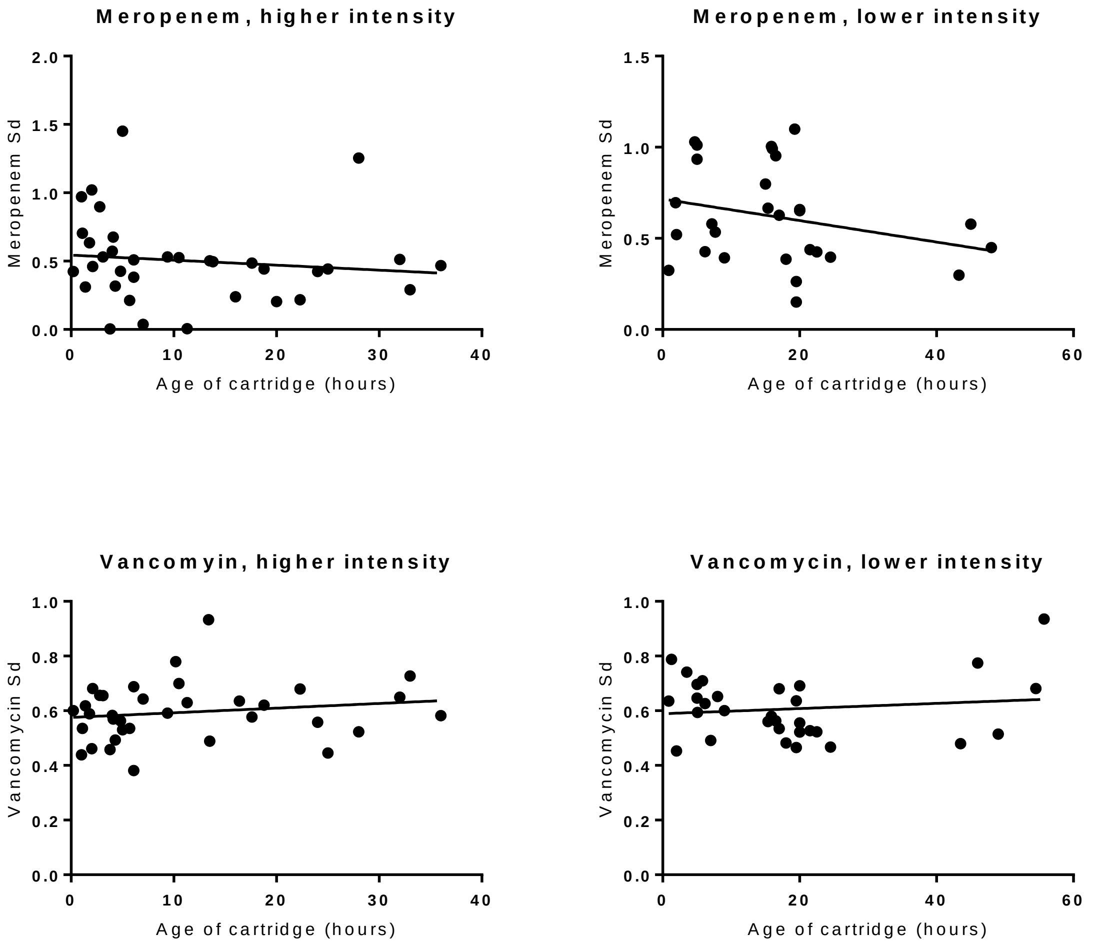

	Age of filter (hours) vs. Meropenem Sd (higher)	Age of filter (hours) vs. Vancomycin Sd (higher)	Age of filter (hours) vs. Meropenem Sd (lower)	Age of filter (hours) vs. Vancomycin Sd (lower)
Pearson r	-0.1206	0.1647	-0.2710	0.1318
95% confidence interval	-0.4363 to 0.2216	-0.1784 to 0.4720	-0.5850 to 0.1135	-0.2335 to 0.4644
r^2	0.01454	0.02711	0.07346	0.01736
P value (two-tailed)	0.4902	0.3445	0.1630	0.4798
Number of XY Pairs	35	35	28	31

Online supplement fig 6. No correlation between the saturation coefficient (Sd) of urea or creatinine with that of meropenem.*

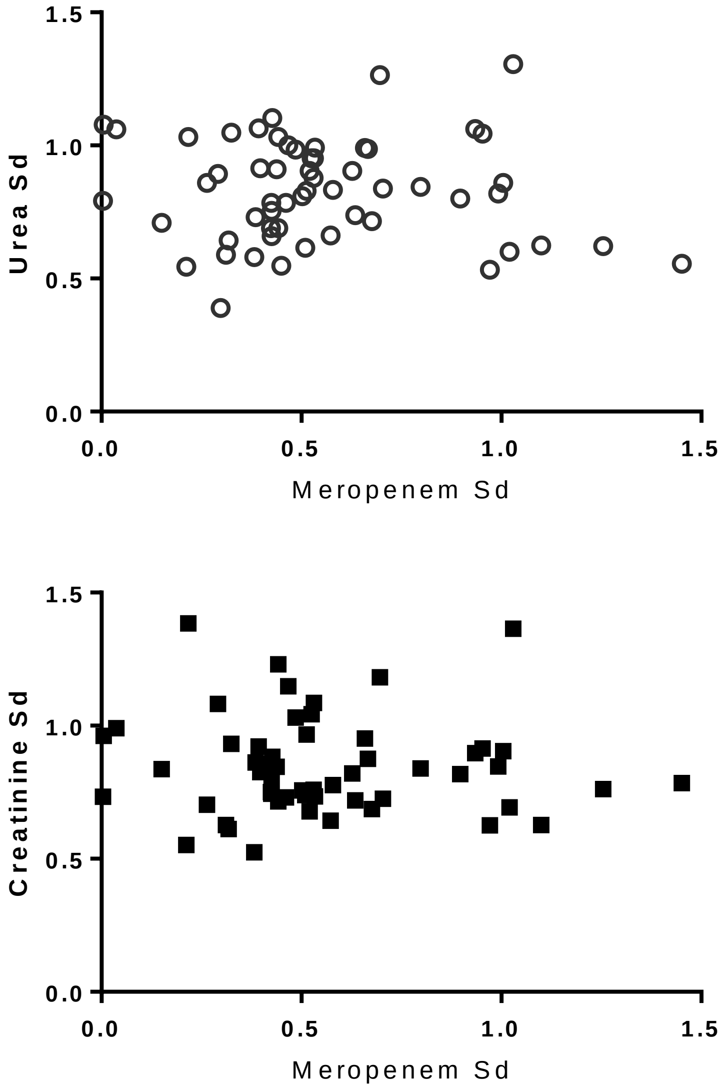

*Urea: Spearman r=-0.0008427, P=0.9950, n=57 pairs; creatinine: Spearman r = -0.01941, P=0.8882, n=55 pairs.

Online supplement fig 7. No correlation between the effluent production rate and the systemic clearance for meropenem# or vancomycin.*

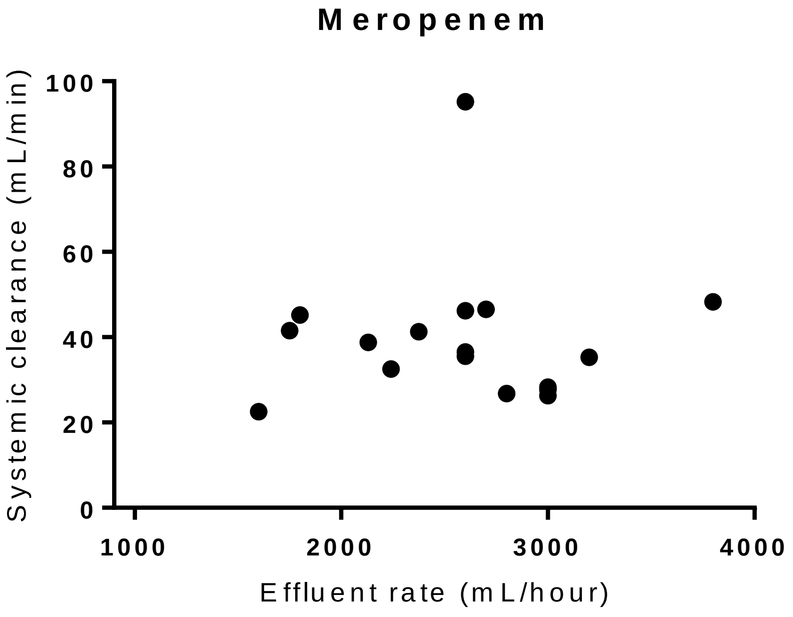

Spearman r = - 0.07417, P = 0.7510

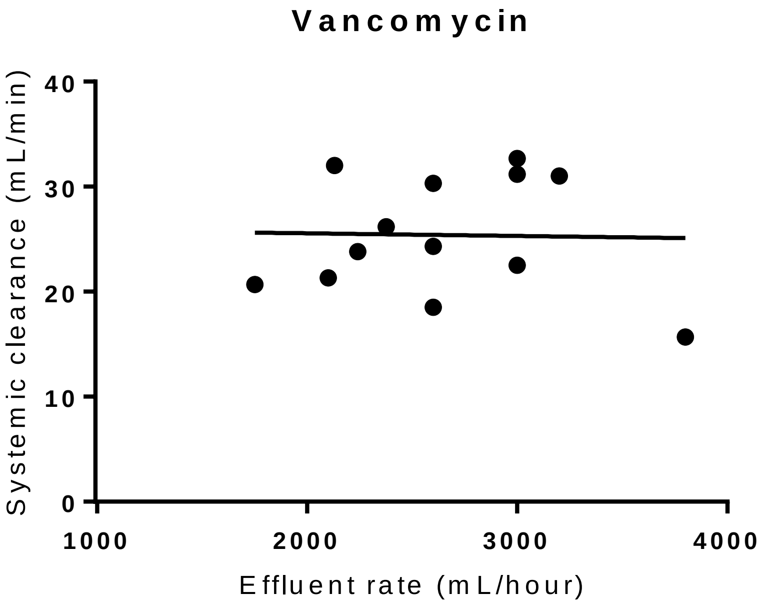

* Pearson r = - 0.02478, r^2 = 0.0006141, P = 0.9360

References

1. Bellomo R, Cass A, Cole L, Finfer S, Gallagher M, Lo S, McArthur C, McGuinness S, Myburgh J, Norton R, et al: **Intensity of continuous renal-replacement therapy in critically ill patients.** *N Engl J Med* 2009, **361:**1627-1638.
2. Wong G, Briscoe S, Adnan S, McWhinney B, Ungerer J, Lipman J, Roberts JA: **Protein binding of beta-lactam antibiotics in critically ill patients: can we successfully predict unbound concentrations?** *Antimicrob Agents Chemother* 2013, **57:**6165-6170.
